# Maternal morbidity associated with violence and maltreatment from husbands and in-laws: findings from Indian slum communities

**DOI:** 10.1186/s12978-016-0223-z

**Published:** 2016-09-08

**Authors:** Jay G. Silverman, Donta Balaiah, Julie Ritter, Anindita Dasgupta, Sabrina C. Boyce, Michele R. Decker, D. D. Naik, Saritha Nair, Niranjan Saggurti, Anita Raj

**Affiliations:** 1 Division of Global Public Health, Department of Medicine, Center on Gender Equity and Health, University of California, San Diego School of Medicine, 9500 Gilman Drive #0507, La Jolla, CA 92093-0507 USA; 2 National Institute for Research on Reproductive Health, Indian Council on Medical Research, Mumbai, India; 3 Department of Population, Family and Reproductive Health, Johns Hopkins Bloomberg School of Public Health, Baltimore, MD USA; 4 Population Council, New Delhi, India

**Keywords:** Intimate partner violence, In-law violence, Gender-based household maltreatment, Maternal health, Maternal morbidity

## Abstract

**Background:**

Intimate partner violence (IPV) victimization is linked to a broad range of negative maternal health outcomes. However, it is unclear whether IPV is directly related to poor maternal outcomes or whether IPV is a marker for other forms of chronic, mundane maltreatment of women that stem from the culture of gender inequity that also gives rise to IPV. To determine the prevalence of non-violent forms of gender-based household maltreatment by husbands and in-laws (GBHM), and violence from in-laws (ILV) and husbands (IPV) against women during the peripregnancy period (during and in the year prior to pregnancy); to assess relative associations of GBHM, ILV and IPV with maternal health.

**Methods:**

Cross-sectional data were collected from women <6 months postpartum (*n* = 1,039, ages 15-35 years) seeking child immunization in Mumbai, India. Associations of IPV, ILV and GBHM during the peripregnancy period with maternal health (prenatal care in first trimester, no weight gain, pain during intercourse, high blood pressure, vaginal bleeding, premature rupture of membranes, premature birth) were evaluated.

**Results:**

One in three women (34.0 %) reported IPV, 4.8 % reported ILV, and 48.5 % reported GBHM during the peripregnancy period. After adjusting for other forms of abuse, IPV related to pain during intercourse (AOR = 1.79); ILV related to not receiving first trimester antenatal care (AOR = 0.49), and GBHM remained associated with premature rupture of membranes (AOR = 2.28), pain during intercourse (AOR = 1.60), and vaginal bleeding (AOR = 1.80).

**Conclusion:**

After adjusting for ILV and IPV, peripregnancy GBHM remained significantly associated with multiple forms of maternal morbidity, suggesting that GBHM is a prevalent and reliable indicator of maternal health risk.

## Plain English summary

This study sought to determine the prevalence of women’s experiences of non-violent, gender-based household maltreatment from their husbands and in-laws (GBHM) during and in the year prior to pregnancy, as well as the prevalence of experiences of overt violence from in-laws (ILV) and husbands (IPV) during these same periods. The relative associations of all three of these forms of abuse to major maternal health outcomes were also assessed.

These data were collected via a survey among women <6 months postpartum (*N* = 1,039, ages 15–35 years) who were seeking child immunization in government clinics in Mumbai, India. Maternal health outcomes assessed included receipt of antenatal care in the first trimester, lack of weight gain during pregnancy, reports of pain during intercourse in pregnancy, high blood pressure in pregnancy, vaginal bleeding in pregnancy, premature rupture of membranes, and premature birth.

We found that 1 in 3 women (34.0 %) reported IPV, 4.8 % reported ILV, and almost half (48.5 %) reported GBHM during and in the year prior to pregnancy. When all three forms of abuse were included in statistical models, IPV remained related to pain during intercourse (adjusted odds ratio = 1.79; AOR), ILV remained related to not receiving first trimester antenatal care (AOR = 0.49), and GBHM remained significantly related to pain during intercourse (AOR = 1.60), vaginal bleeding (AOR = 1.80), and premature rupture of membranes (AOR = 2.28). We conclude that GBHM is both a highly prevalent form of abuse, and that assessment of GBHM may assist practitioners to identify those women at highest maternal health risk.

## Background

Based, in part, on the establishment of the Millennium Development Goals (MDGs) 4 and 5 to reduce child mortality and improve maternal health, respectively, the past two decades have been characterized by a heightened focus on reducing neonatal and childhood morbidity across India, which has included a ‘safe motherhood’ model that has encouraged institutional deliveries and antenatal care starting in the first trimester [1–3]. Despite these efforts, India has fallen short of the benchmarks set for both of these development goals [1]. Based on the most recent nationally-representative data, less than half (43 %) of pregnant women in India received antenatal care in the first trimester in the years 2005 and 2006 [4]. In addition to insufficient antenatal care, women in India continue to experience over 3.5 million preterm births, accounting for more than 1 in 8 births nationally and 1 in 4 preterm births globally [5]. Relatedly, large numbers of women in India have low maternal body mass index [6] and low gestational weight gain during pregnancy [7] outcomes which have been linked to low birthweight [6, 8] and maternal and neonatal death [1, 9]. High blood pressure in pregnancy, specifically hypertensive disorders of pregnancy (HPD), are estimated to account for approximately half of complications during pregnancy among women in India seeking medical care for such concerns [10]. HPD is also linked to excessive vaginal bleeding, preterm birth and low birthweight [11, 12].

Multiple recent studies have found that maternal intimate partner violence (IPV) victimization is also consistently linked to a broad range of negative maternal and child health outcomes, including not receiving antenatal care in the first trimester, lack of weight gain during pregnancy, preterm birth and low birth weight [13–17]. More than 1 in 3 women ages 15-49 in India report IPV from their current partner; notably, pregnancy is not a protective period regarding IPV in this national context, with studies of IPV during and immediately following pregnancy estimating prevalence of 15–26 % [18].

Given that IPV appears to be linked to the health and health behaviors of women during pregnancy, it is unclear whether IPV is *directly related* to poor maternal outcomes or whether IPV is a *marker* for other forms of chronic, mundane, non-physically violent maltreatment of women that stem from the culture of gender inequity that also gives rise to IPV, and that are instrumental in increasing maternal health risk (e.g., preventing access to antenatal care and adequate nutrition).

In addition to violence and maltreatment from husbands, women in India are also vulnerable to abuse from their in-laws. The traditional family structure in India involves women moving after marriage to her husband’s community and co-residing with his parents and family. In this patrilocal cultural context, in-laws may be a source of protection or a source of violence and health risk for a woman [19, 20]. This may be independent of or in conjunction with violence and maltreatment from husbands; [19, 20] a recent study found that Indian women experiencing IPV during pregnancy or within 6 months postpartum were over five times more likely to report in-law violence (ILV) during this same period [21].

Previous qualitative research by the authors of the current study, conducted among women in India who had recently given birth and who reported experiencing IPV during their recent pregnancy, generated a compendium of common non-violent forms of abuse from husbands and in-laws that were described, in themselves, as compromising the health of these women [21]. This compendium included, but was not limited to, limiting nutrition, prohibiting periods of rest, limiting access to medical care for them or their child, and coerced heavy domestic labor [21]. These behaviors were labeled gender-based household maltreatment (GBHM), and prior analyses indicate that these forms of abuse are more common than overt physical violence from in-laws or husbands [21]. Given the high prevalence of IPV against women in India, [3] the great need to reduce the continuing high levels of maternal and infant morbidity and mortality in this context, and the consistent associations observed between IPV and these critical outcomes, it is essential to understand how IPV and other forms of gender-based maltreatment relate to maternal and child health in order to make progress towards global development goals.

Specifically, in order to advance the current state of knowledge regarding the roles of gender-based violence and maltreatment from both husbands and in-laws in compromising maternal health, the current study assessed the relative prevalence and overlap of GBHM, IPV and ILV during the peripregnancy period (during or in the year prior to pregnancy), and the independent associations of these forms of abuse with common forms of maternal morbidity. Findings regarding the relative influence of these different forms of gender-based violence and maltreatment will provide guidance for development of programs and policies that attempt to improve maternal health through identification and addressing factors at the household level.

## Methods

Cross-sectional, quantitative data were collected from women (ages 15–35) seeking immunizations for their infants <6 months of age between August and December 2008. Recruitment and data collection occurred at three large urban health centers (UHCs), selected based on their serving more than 100,000 residents in each of three major slum communities in Mumbai, India. Prior research documents very high rates of infant immunization (97 %) in Mumbai slum areas [16], allowing recruitment from a sample likely generalizable to the larger population. Women were approached subsequent to their receiving immunizations for their children to determine whether they met the following eligibility criteria: a) having an infant <6 months of age and b) being willing to learn more about a study examining conflict in the family and health issues for women and children. All potential participants were led to a private room within the clinic where informed consent forms were read aloud due to concerns regarding low literacy of participants. Those providing verbal informed consent then completed a quantitative survey with a trained, female research staff member from the Indian National Institute of Research in Reproductive Health (NIRRH) reading all questions aloud and recording the answers provided on a paper survey form. All staff members were trained in research ethics, data collection, and interviewing women experiencing IPV. The survey required between 30–40 min to complete and was conducted in Marathi (the native language of Maharashtra) or Hindi, based on participant preference; survey items were developed first in English, then translated to Marathi and Hindi, and then back-translated to English to assure fidelity to original content. Following survey completion, all participants were screened for emotional distress and were given resources for legal, mental health and IPV-related assistance. Institutional review boards at the University of California at San Diego School of Medicine and the Indian Council of Medical Research approved all study procedures.

During the recruitment period, a total of 1,830 women were approached sequentially for screening. All women presenting to the clinic seeking infant vaccinations were found to be eligible based on their having an infant < 6 months of age. Of these women, 60.5 % (1,108/1,830) agreed to meet privately with a research team member to learn more about the study; the major reason provided for not agreeing to hear more about the study was lack of time. Of women agreeing to hear about the study, 94.6 % (1,049/1,108) provided consent and completed the survey.

### Measures

Demographics of women were assessed via single-item measures and included age, completion of any formal education, household income, religion, family structure (nuclear or co-residing with in-laws), age at marriage, and number of children. Husband characteristics assessed via report of female participants included husband’s age relative to the woman’s age (i.e., how many years older was the husband) and completion of any formal education.

#### Violence and gender-based household maltreatment

Peripregnancy IPV, violence from in-laws and gender-based household maltreatment measures were developed based on findings of previous research and the Indian Demographic and Health Surveys (a.k.a., Indian National Family Health Survey-2) [21, 22]. All abuse-related items used for the measurement of each of these variables were assessed dichotomously (i.e., yes/no), and separately for the year prior to the most recent pregnancy and during this pregnancy. Peripregnancy IPV was measured via four items specific to each of the assessed periods (year prior to pregnancy and during pregnancy): (1) “Did your husband hit, push, kick, beat, or slap you?” (2) “Did your husband try to burn you?” (3) “Did your husband insist on sex when you did not want to have sex?” (4) “Did your husband use force to make you have sex when you did not want to have sex?” If they said yes to any of these four items for either of the two periods, they were defined as having experienced peripregnancy IPV. Cronbach’s alpha for this 8-item measure was 0.78.

Peripregnancy violence from in-laws (ILV) was measured via two items for each of the two assessed peripregnancy periods: (1) “Did your in-laws hit, push, kick, beat, or slap you?” (2) “Did your in-laws try to burn you?” If they said yes to either of these items for either period assessed, they were defined as having experienced peripregnancy ILV. Cronbach’s alpha for this 4-item measure was 0.79.

Peripregnancy GBHM was defined as non-violent forms of abuse from husbands or in-laws occurring either in the year prior to the most recent pregnancy, or during the pregnancy. As previously discussed, this measure was created based on formative qualitative research with postpartum women drawn from this same population. The GBHM scale assessed for 12 forms of this type of abuse; 10 GBHM items were asked separately regarding abuse specific to husbands and to in-laws and, as with the violence measures, for both the year prior to pregnancy and during pregnancy (with the exception of one item, as noted below). These items included “Did your (husband/in-laws) force you to bring money or other things from your parents’ home?”, “Did your (husband/in-laws) interfere in your ability to get health care for yourself?”, “Did your (husband/in-laws) interfere in your ability to get health care for your children?”, “Did your (husband/in-laws) stop you from getting enough food for yourself?”, “Did your (husband/in-laws) stop you from getting enough food for your children?”, “Did your (husband/in-laws) stop you from getting the rest you needed?”, “Did your (husband/in-laws) attempt to stop you from going to your natal home for the birth?” (not assessed for year prior to pregnancy period), “Did your (husband/in-laws) treat you badly for not having a boy child?”, “Did your (husband/in-laws) stop you from taking care of your children?”, “Did your (husbands/in-laws) neglect/ignore your baby?” An additional GBHM item was asked only of husbands for the year prior to pregnancy and during pregnancy periods: “Did you ever feel that you needed help to care for your elder children from your husband but didn’t receive it?” Two forms of GBHM were not specific to either husbands or in-laws; these related to burdens of household labor during the year prior to pregnancy or during pregnancy: “Did anyone assist you to prepare meals for the household?”, “Did anyone assist you to perform cleaning work for the household?” Participants responding “yes” to one or more of the husband/in-law items or “no” to either of the household work items, either during pregnancy or post-partum, were coded as having experienced peripregnancy partner or in-law GBHM. The final 44-item measure had a Cronbach alpha of 0.95. (NOTE: To test for collinearity, correlations were assessed among the main predictor variables [IPV, ILC, husband GBHM and in-law GBHM]; the correlation between husband and in-law GBHM exceeded *r* = 0.70 and, for this reason, husband and in-law GBHM were considered as a single variable in all subsequent analyses.)

#### Maternal health outcomes

Self-report items from the core Demographic and Health Surveys (DHS) were utilized to assess whether or not women experienced the following health outcomes during their recent pregnancy: *antenatal care in the first trimester, no weight gain, high blood pressure, pain during intercourse, vaginal bleeding, premature rupture of membranes, and premature birth* [23].

### Data analyses

Descriptive statistics were generated for all variables, for both the total sample and by each maternal health outcome. Chi-square tests were used to evaluate the associations between socio-demographic characteristics and each maternal health outcome (*p* < 0.05; see Table 1). Logistic regression models were then constructed to assess the associations between abuse variables (peripregnancy IPV, ILV, and GBHM) and these outcomes (*p* < 0.05). In addition to including all three forms of abuse, adjusted models were constructed with consideration of covariates previously documented as affecting maternal health risk; these included maternal age, education, religion, household income, husband >5 years older than wife, husband’s education, family type (nuclear vs. co-residing with in-laws), and parity (number of children prior to index pregnancy). Covariates were considered for inclusion in models based on having a bivariate association with the given health outcome at *p* < 0.30. Logistic regression models were then refined using a backwards stepwise selection process with *p* < 0.05 as the inclusion criteria [24]. Abuse variables (peripregnancy IPV, ILV, and GBHM) and women’s age were retained in all adjusted models based on aims of the current study and known importance of maternal age regarding health during pregnancy. All analyses were conducted using SAS software, Version 9.2 (Cary, NC, USA).

**Table 1:** Sample demographics and prevalence of pregnancy related maternal health outcomes among women in Mumbai, Maharashtra, India (*N* = 1,049^a^)

	Total (*N* = 1,049) % (n)	Prenatal care in first trimester (46.3 %, *n* = 485) % (n)	No weight gain (8.1 %, *n* = 83) % (*n*)	Pain during intercourse (15.6 %, *n* = 163) % (n)	High blood pressure (11.2 %, *n* = 113) % (n)	Vaginal bleeding (8.8 %, *n* = 92) % (n)	Premature rupture of membranes (10.8 %, *n* = 113) % (n)	Premature birth (13.8 %, *n* = 144) % (n)
Age								
< 20 years	7.6 (81)	63.0 (51)^*^	8.8 (7)	19.7 (16)^**^	11.1 (9)^**^	7.4 (6)	9.9 (8)	22.2 (18)^**^
20–24 years	48.1 (504)	53.5 (261)	8.7 (43)	15.7 (79)	9.5 (46)	9.6 (48)	11.4 (57)	13.2 (66)
25–29 years	29.4 (308)	38.7 (115)	7.3 (22)	16.6 (51)	11.8 (35)	7.5 (23)	9.4 (29)	12.1 (37)
> 30 years	14.9 (156)	40.0 (58)	7.2 (11)	11.0 (17)	16.0 (23)	9.7 (15)	12.3 (19)	14.9 (23)
Any Formal Education								
Yes	84.2 (883)	50.9 (440)^*^	8.0 (70)	15.9 (140)	11.3 (97)	8.7 (77)	10.1 (89)^**^	15.2 (134)^*^
No	15.7 (166)	30.6 (45)	8.2 (13)	14.1 (23)	10.8 (16)	9.2 (15)	14.6 (24)	6.1 (10)
Religion								
Hindu	37.7 (395)	52.9 (203)^*^	6.7 (26)	11.5 (45)^*^	10.0 (38)	7.4 (29)	8.6 (34)^**^	13.0 (51)
Muslim	58.9 (618)	44.0 (260)	9.1 (55)	18.7 (115)	11.9 (70)	9.7 (60)	12.5 (77)	14.0 (86)
Other	3.4 (36)	61.1 (22)	5.6 (2)	8.3 (3)	13.9 (5)	8.3 (3)	5.6 (2)	19.4 (7)
Household income (rupees)								
< =3,000	23.2 (243)	37.0 (87)^*^	12.6 (30)^*^	14.9 (36)	14.0 (33)	12.8 (31)^*^	12.8 (31)	14.5 (35)^*^
> 3,000 and < =4,500	24.9 (261)	44.7 (109)	10.8 (27)	14.2 (37)	9.4 (23)	9.2 (24)	9.2 (24)	9.6 (25)
> 4,500 and < =7,000	27.0 (283)	52.2 (143)	4.6 (13)	17.4 (49)	8.2 (22)	6.0 (17)	9.9 (28)	13.1 (37)
> 7,000	24.8 (260)	56.6 (146)	5.0 (13)	15.8 (41)	13.6 (35)	7.7 (20)	11.5 (30)	18.1 (47)
Husband’s relative age to wife’s age								
< 5 years older	56.0 (587)	44.2 (259)^**^	8.8 (51)	15.9 (93)	11.3 (64)	8.0 (47)^**^	11.6 (68)	14.7 (86)^**^
5–10 years older	41.4 (434)	48.7 (211)	7.1 (30)	15.1 (65)	11.4 (47)	10.4 (45)	9.7 (42)	11.8 (51)
> 10 years older	2.7 (28)	53.6 (15)	7.4 (2)	17.9 (5)	7.7 (2)	0.0 (0)	10.7 (3)	25.0 (7)
Husband Any Formal Education								
Yes	87.6 (919)	50.2 (446)^*^	8.2 (74)	16.0 (146)	11.8 (104)^**^	9.1 (83)	10.9 (100)	14.3 (131)^**^
No	12.4 (130)	31.7 (39)	7.3 (9)	13.2 (17)	7.3 (9)	7.0 (9)	10.1 (13)	10.1 (13)
Family Type								
Nuclear	38.5 (403)	42.9 (164)^*^	6.4 (25)^**^	12.0 (48)^*^	10.0 (38)	7.2 (29)^**^	6.7 (27)^*^	11.0 (44)^*^
Joint	61.6 (645)	51.0 (321)	9.1 (58)	17.9 (115)	11.9 (75)	9.8 (63)	13.4 (86)	15.5 (100)
Number of Other Children								
0	40.4 (422)	58.1 (245)^*^	8.6 (36)^**^	17.3 (73)^**^	11.8 (49)^**^	10.2 (43)^**^	13.3 (55)^**^	16.6 (70)^**^
1	32.4 (338)	45.0 (152)	9.4 (31)	16.0 (54)	8.2 (27)	6.8 (23)	9.2 (31)	11.3 (38)
2–3	22.9 (239)	31.8 (76)	4.8 (11)	13.9 (33)	13.5 (30)	10.5 (25)	9.6 (23)	12.2 (29)
4+	4.3 (45)	26.7 (12)	11.4 (5)	4.4 (2)	15.4 (6)	2.2 (1)	4.4 (2)	15.6 (7)

^a^All outcomes had less than 5 % missing responses and each bivariate analysis had less than with a missing response to the outcome or demographic variable

^*^Significant differences between groups observed (*p* < 0.05) and considered for inclusion in multivariate models

^**^Considered for inclusion in multivariate models (*p* < 0.30)

## Results

### Peripregnancy intimate partner violence (IPV), violence from in-laws (ILV), and gender-based household maltreatment (GBHM)

**Fig. 1:**
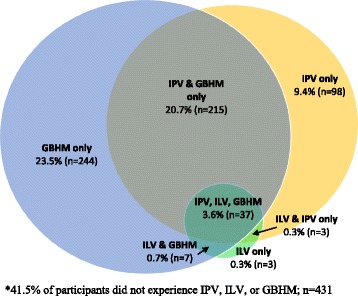
Husband intimate partner violence, in-law violence and gender-based household maltreatment during pregnancy and post-natal period

More than one in three women (34.0 %) reported IPV in the year prior to and/or during their latest pregnancy (9.7 % year prior to pregnancy only, 4.3 % during pregnancy only, 19.9 % both prior to and during pregnancy). A smaller number (4.8 %) reported violence from in-laws in the same time period (2.4 % year prior to pregnancy only, 0.6 % during pregnancy only, 1.8 % both prior to and during pregnancy). Approximately one half of women (48.5 %) reported one or more forms of peripregnancy GBHM (4.8 % year prior to pregnancy only, 4.9 % during pregnancy only, 38.7 % both prior to and during pregnancy). Regarding the co-occurrence of these forms of abuse, ILV almost completely co-occurred with GBHM, IPV, or both (94.0 %); the majority (71.4 %) of cases of IPV also include GBHM. However, about half (48.5 %) of cases of GBHM occurred in absence of either IPV or ILV (see Fig. 1).

The most common forms of GBHM were not receiving assistance from family with household work (cleaning and meal preparation), both in the year prior to pregnancy (35.1 % and 37.8 %, respectively) and during pregnancy (29.5 % and 32.4 %, respectively). Being forced by husbands or in-laws to demand money from their natal family either in the year prior to (3.6 % and 5.0 %, respectively) or during pregnancy (3.7 % and 4.7 %, respectively), husband or in-laws preventing women from getting adequate rest in the year prior to pregnancy (3.9 % and 5.3 %, respectively) and during pregnancy (4.1 % and 5.2 %), and husband or in-laws preventing women from going to their natal homes during pregnancy (10.6 % and 8.3 %) were also reported. Slightly less common forms of GBHM reported were in-laws preventing women from getting adequate food during pregnancy (3.0 %), and interference from husbands or in-laws in women’s ability to seek health care during pregnancy (2.1 % and 2.5 %).

### Associations of peripregnancy IPV, ILV and GBHM with maternal health

In unadjusted analyses, significant associations were seen for reduced likelihood of receiving antenatal care during the first trimester based on experiences of IPV, ILV and GBHM (ORs 0.40–0.66) (Table 2). Similarly, all three forms of abuse were found to be associated with no weight gain (ORs 1.68–2.54) and pain during intercourse (ORs 1.67–2.25) during pregnancy. IPV and GBHM were associated with premature rupture of membranes (PROM) (ORs 1.53–1.89). No forms of abuse were associated with either high blood pressure or premature birth.

**Table 2:** Unadjusted associations of peripregnancy abuse (IPV, ILV, and GBHM) and maternal health outcomes, Mumbai, Maharashtra, India (*N* = 1,049)

	Total sample *N* = 1,049 % (n)	Prenatal care in first trimester (*n* = 485) % OR (95 % CI)	No weight gain during pregnancy (*n* = 83) % OR (95 % CI)	Pain during intercourse (*n* = 163) % OR (95 % CI)	High blood pressure (*n* = 113) % OR (95 % CI)	Vaginal bleeding (*n* = 92) % OR (95 % CI)	Premature rupture of membranes (*n* = 113) % OR (95 % CI)	Premature birth (*n* = 144) % OR (95 % CI)
Peripregnancy IPV								
Any	34.0 (353)	41.3	10.7	22.1	12.2	11.1	13.6	14.2
		**0.66 (0.51–0.86)**	**1.68 (1.07–2.65)**	**2.03 (1.45–2.85)**	1.16 (0.77–1.74)	1.48 (0.96–2.29)	**1.53 (1.03–2.28)**	1.08 (0.75–1.57)
None^a^	66.0 (685)	51.4	6.7	12.3	10.7	7.7	9.3	13.3
		-	-	-	-	-	-	-
Peripregnancy ILV								
Any	4.8 (50)	26.0	16.7	28.0	14.6	14.0	18.0	14.0
		**0.40 (0.21–0.76)**	**2.54 (1.16–5.55)**	**2.25 (1.19–4.26)**	1.45 (0.64–3.26)	1.82 (0.81–4.11)	1.96 (0.93–4.11)	1.10 (0.49–2.45)
None^a^	95.2 (988)	47.4	7.6	15.0	11.0	8.6	10.4	13.6
		-	-	-	-	-	-	-
Peripregnancy GBHM								
Any	48.5 (503)	39.4	10.3	19.1	12.8	11.5	13.9	12.3
		**0.58 (0.45–0.74)**	**1.83 (1.16–2.91)**	**1.67 (1.19–2.35)**	1.36 (0.92–2.01)	**1.91 (1.23–2.96)**	**1.89 (1.26–2.82)**	0.81 (0.57–1.16)
None^a^	51.5 (535)	52.9	5.9	12.3	9.8	6.4	7.9	14.8
		-	-	-	-	-	-	-

^a^Referent group is no IPV/ILV/GBHM

All bolded statistics are signficant at *p* < .05

In regression models that included all forms of abuse and were adjusted for all indicated covariates, IPV remained a significant predictor only of pain during intercourse (AOR = 1.79, 95 % CI 1.23–2.60) (Table 3). For antenatal care during the first trimester, only ILV remained a significant predictor in adjusted models (AOR = 0.49, 95 % CI 0.24–0.95). GBHM remained significantly associated with PROM (AOR = 2.28, 95 % CI 1.43–3.63), and pain during intercourse (AOR = 1.60, 95 % CI (1.09–2.34) and vaginal bleeding during pregnancy (AOR = 1.80, 95 % CI 1.11–2.91). No assessed forms of abuse remained significantly associated with no weight gain during pregnancy.

**Table 3:** Adjusted associations of peripregnancy abuse (IPV, ILV, and GBHM) and maternal health outcomes, Mumbai, Maharashtra, India (*N* = 1,049)

	Prenatal care in first trimester (*n* = 485)	No weight gain during pregnancy (*n* = 83)	Pain during intercourse (*n* = 163)	High blood pressure (*n* = 113)	Vaginal bleeding (*n* = 92)	Premature rupture of membranes (*n* = 113)	Premature birth (*n* = 144)
	AOR^a^ (95 % CI)	AOR^a^ (95 % CI)	AOR^a^ (95 % CI)	AOR^a^ (95 % CI)	AOR^a^ (95 % CI)	AOR^a^ (95 % CI)	AOR^a^ (95 % CI)
Peripregnancy IPV							
Any	0.85 (0.63–1.15) ^*^	1.32 (0.79–2.21) ^**^	**1.79 (1.23–2.60)** ^***^	1.03 (0.66–1.61) ^****^	1.19 (0.73–1.92) ^****^	1.36 (0.86–2.15) ^*****^	1.12 (0.74–1.69) ^******^
None^b^	-	-	-	-	-	-	-
Peripregnancy ILV							
Any	**0.48 (0.24–0.95)** ^*^	1.88 (0.80–4.45) ^**^	1.34 (0.67–2.69) ^***^	1.30 (0.55–3.07) ^****^	1.35 (0.57–3.23) ^****^	1.15 (0.49–2.68) ^*****^	1.10 (0.47–2.58) ^******^
None^b^	-	-	-	-	-	-	-
Peripregnancy GBHM							
Any	0.80 (0.60–1.06) ^*^	1.50 (0.89–2.53) ^**^	**1.60 (1.09–2.34)** ^***^	1.27 (0.83–1.94) ^****^	**1.80 (1.11–2.91)** ^****^	**2.28 (1.43–3.63)** ^*****^	0.81 (0.55–1.19) ^******^
None^b^	-	-	-	-	-	-	-

^a^ All models include IPV, ILV, GBHM, and age

^b^ Referent is no IPV/ILV/GBHM

^*^ Additional significant covariates: education, household income, husband’s relative age to wife’s age, husband formal education, number of other children (*p* < 0.05)

^**^ Additional significant covariates: household income, family type (*p* < 0.05)

^***^ Additional significant covariates: family type (*p* < 0.05)

^****^ No additional significant covariates (*p* < 0.05)

^*****^ Additional significant covariates: religion, family type, number of other children (*p* < 0.05)

^******^ Additional significant covariate: education (*p* < 0.05)

All bolded statistics are signficant at *p* < .05

## Discussion

Both IPV and GBHM are prevalent peripregnancy concerns among women in India. Results of the current study indicate that more than one-third of women experience violence from their husbands during either the year prior to pregnancy or during pregnancy, with the majority reporting experiencing such violence across both periods. Even more prevalent is GBHM, studied for the peripregnancy period for the first time via the current study. Approximately one-half of women reported these forms of maltreatment from husbands and in-laws across at least one of these periods, and more than one-third of them reported GBHM across both the year prior to pregnancy and during pregnancy. Also new to the literature, violence from in-laws during the year prior to pregnancy and during pregnancy was estimated at 5 %.

All three forms of gender-based peripregnancy abuse were found to be associated with a subset of maternal health outcomes. Importantly, in this first study of in-law violence and maternal health, peripregnancy violence from in-laws was the single form of abuse observed that remained significantly associated with women not receiving timely antenatal care, a major risk factor for both maternal and infant mortality [25, 26]. This finding is consistent with previous qualitative research in this setting indicating that mothers-in-law will sometimes interfere with and actively block access to health services by their daughters-in-law [21]. Further, in this first study of GBHM and maternal health, GBHM was found to be associated with the same forms of poor maternal health that were associated with IPV. After adjusting for the other forms of abuse included in the current study, peripregnancy GBHM remained significantly associated with pain during intercourse, vaginal bleeding and PROM. In contrast, peripregnancy IPV remained associated with one of these same outcomes, pain during intercourse, and no other indicators of poor maternal health. These findings suggest that GBHM is not only more prevalent than IPV, but also may be a more reliable indicator of maternal health risk. Implications for maternal health promotion and clinical practice are discussed below.

Neither IPV, ILV, nor GBHM were found to be associated with high blood pressure during pregnancy or premature delivery. Previous studies of IPV and maternal health have yielded mixed findings, with one identifying associations between violence from male partners during the year prior to pregnancy and both pregnancy-related high blood pressure and premature delivery, but not during pregnancy, [27] and another study finding that IPV during pregnancy was associated with preterm delivery [28].

Although correlated, GBHM and IPV were not collinear, with half of women reporting GBHM not reporting IPV (Fig. 1). The implications of these findings for maternal health promotion and antenatal practice are that screening for and addressing IPV in these contexts, although of clear utility to identify those at risk for continued abuse, may not be as effective as screening for GBHM as a protocol to identify those women at greatest risk for poor maternal health. Further, as GBHM behaviors are normative household phenomena and not stigmatized, addressing and reducing GBHM, relative to IPV, may be both more feasible and acceptable within existing community-based maternal health promotion and care structures (e.g., community health worker visits to households of pregnant women to promote proper nutrition, antenatal care and institutional delivery).

The current findings should be viewed in the context of several significant limitations related to the design of the study. The self-report nature of outcomes may have led to over or under-reporting of these phenomena, the reasons for which may include fear of retaliation and/or normalization of such maltreatment, and may have been influenced by social desirability bias. The study design was cross-sectional, precluding assumptions of causality or directionality. Also, the current sample represents only women residing in a small number of low-income urban communities in Mumbai, India; thus, the findings currently described may not hold true across other geographic and socioeconomic contexts. As noted earlier, approximately 40 % of those who were approached for study recruitment declined participation, most often due to a lack of time. Although the demographic profile of the current sample matched that of overall immunization clinic attended based on clinic registration, it is possible that those who chose to participate in the study were otherwise systematically different from those who refused, e.g., they may have reported having less time due to fears of abuse related to being absent from the household for a longer period. Such bias would lead to a current underestimation of the prevalence of forms of abuse. Finally, this study focuses on a new construct and related assessment – GBHM. While GBHM is intended to represent non-violent forms of abuse, it is not possible to determine whether or not physical or sexual violence coincided with these incidents of maltreatment. Further research will be required to extend this initial validation of both the construct and measure.

## Conclusions

Findings from the current study provide initial data regarding the question of whether GBHM (or other forms of gender-based non-violent maltreatment at the household level) operationally and mechanistically lead to multiple threats to maternal and child morbidity and mortality in the Indian context, and do so to a greater extent than IPV, based on IPV potentially being a marker for a household climate of high levels of gender inequity that devalues women and girls. Testing of this hypothesis will require research that includes larger, representative samples across national contexts, and longitudinal study to clarify chronology and roles of violence and non-violent abuse regarding maternal and child health.

## Abbreviations

AOR: Adjusted odds ratio
DHS: Demographic and health surveys
GBHM: Gender-based household maltreatment
HPD: Hypertensive disorders of pregnancy
ILV: In-law violence
IPV: Intimate partner violence
MDG: Millennium development goals
NFHS: National family health survey
NIRRH: Indian National Institute of Research in Reproductive Health
PROM: Premature rupture of membranes
UHC: Urban health centers
